# Sequential Activation of Classic PKC and Estrogen Receptor α Is Involved in Estradiol 17ß-D-Glucuronide-Induced Cholestasis

**DOI:** 10.1371/journal.pone.0050711

**Published:** 2012-11-27

**Authors:** Ismael R. Barosso, Andrés E. Zucchetti, Andrea C. Boaglio, M. Cecilia Larocca, Diego R. Taborda, Marcelo G. Luquita, Marcelo G. Roma, Fernando A. Crocenzi, Enrique J. Sánchez Pozzi

**Affiliations:** Instituto de Fisiología Experimental (IFISE), Facultad de Ciencias Bioquímicas y Farmacéuticas (CONICET – U.N.R.), Rosario, Argentina; Thomas Jefferson University, United States of America

## Abstract

Estradiol 17ß-d-glucuronide (E17G) induces acute cholestasis in rat with endocytic internalization of the canalicular transporters bile salt export pump (Abcb11) and multidrug resistance-associated protein 2 (Abcc2). Classical protein kinase C (cPKC) and PI3K pathways play complementary roles in E17G cholestasis. Since non-conjugated estradiol is capable of activating these pathways *via* estrogen receptor alpha (ERα), we assessed the participation of this receptor in the cholestatic manifestations of estradiol glucuronidated-metabolite E17G in perfused rat liver (PRL) and in isolated rat hepatocyte couplets (IRHC). In both models, E17G activated ERα. In PRL, E17G maximally decreased bile flow, and the excretions of dinitrophenyl-glutathione, and taurocholate (Abcc2 and Abcb11 substrates, respectively) by 60% approximately; preadministration of ICI 182,780 (ICI, ERα inhibitor) almost totally prevented these decreases. In IRHC, E17G decreased the canalicular vacuolar accumulation of cholyl-glycylamido-fluorescein (Abcb11 substrate) with an IC50 of 91±1 µM. ICI increased the IC50 to 184±1 µM, and similarly prevented the decrease in the canalicular vacuolar accumulation of the Abcc2 substrate, glutathione-methylfluorescein. ICI also completely prevented E17G-induced delocalization of Abcb11 and Abcc2 from the canalicular membrane, both in PRL and IRHC. The role of ERα in canalicular transporter internalization induced by E17G was confirmed in ERα-knocked-down hepatocytes cultured in collagen sandwich. In IRHC, the protection of ICI was additive to that produced by PI3K inhibitor wortmannin but not with that produced by cPKC inhibitor Gö6976, suggesting that ERα shared the signaling pathway of cPKC but not that of PI3K. Further analysis of ERα and cPKC activations induced by E17G, demonstrated that ICI did not affect cPKC activation whereas Gö6976 prevented that of ERα, indicating that cPKC activation precedes that of ERα. Conclusion: ERα is involved in the biliary secretory failure induced by E17G and its activation follows that of cPKC.

## Introduction

Bile secretion is a complex and regulated process that finally depends on the activity of transporters located in the canalicular pole of the hepatocyte that mainly belong to the ABC superfamily of ATP-dependent transporters [1], [2]. Among the most relevant transporters involved in bile formation are the *bile salt export pump* (Abcb11, also named Bsep), which transports monoanionic bile salts, and the *multidrug-resistance associated protein 2* (Abcc2, also named Mrp2), which transports glutathione and glutathione conjugates, as well as a wide variety of anionic compounds [1], [2]; bile salts and glutathione are chief determinants of the so called bile salt-dependent and bile salt-independent fractions of bile flow, respectively [3]. Alterations of canalicular transporter expression, localization, or activity can lead to cholestasis [4].

Estradiol 17ß-d-glucuronide (E17G) is a D-ring endogenous metabolite of estradiol that induces acute and reversible cholestasis *in vivo*, by impairing both fractions of bile flow [5]. The levels of D-ring metabolites increase during pregnancy [6] and may be a key factor in the pathogenesis of intrahepatic cholestasis occurring in pregnant, susceptible women [5]. As a likely mechanism, E17G induces microtubule-independent endocytic internalization of both Abcb11 [7] and Abcc2 [8], [9].

Intracellular signaling has emerged as a fundamental fact to explain the development of different models of cholestasis [10]–[14]. In E17G induced cholestasis, our group demonstrated the activation of both the “classical” (Ca^2+^-dependent) PKC isoforms (cPKC) [12], and the PI3K-Akt pathway [13]. The blockage of either of these pathways partially prevented cholestasis whereas the inhibition of both together almost completely prevented E17G actions. This indicates that E17G activates at least two branches of signaling pathways to achieve cholestasis. Hence, it was of interest to investigate other potential signaling proteins to build the cascade that leads from E17G initial action to cholestasis passing through cPKC and PI3K activation.

It is remarkable that non-conjugated estradiol is also capable of activating cPKC and PI3K in different tissues including the liver [15], [16]. Estrogen actions can be divided in cytosolic (non-genomic) and nuclear (genomic) [17]. Cytosolic actions are stimulated in short time and can participate in E17G cholestasis, which is acute in nature. Several proteins have been implicated in estradiol rapid cytosolic effects [17], among them it should be mentioned the estrogen receptor (ER). This protein has two isoforms ERα and ERß, although only the first is present in the liver [18]. In line with this, Tollefsen et al. demonstrated that E17G interacts to fish hepatic estrogen receptor with low affinity [19] and given that the ligand-binding regions are moderately conserved it is possible that E17G potentially interacts with ERα. The interaction of E17G with ERα could be considered a logical consequence of its estrogen nature; however, there is no previous study on the role of the receptor in E17G cholestasis. Besides, it is known that ERα shares signaling pathways with cPKC or PI3K-Akt [15], [16] and, therefore, it is of interest to place the eventual participation of ERα in the context of the other signaling molecules that were demonstrated to be involved in E17G-induced cholestasis. Hence, the aim of this work is to prove the role of ERα in the E17G-induced cholestasis and whether there is a connection between ER activation and the pro-cholestatic actions of cPKC and PI3K.

## Materials and Methods

### Materials

Cholyl-glycylamido-fluorescein (CGamF) was kindly provided by Prof. Alan Hofmann (University of California, San Diego). E17G, ICI 182,780 (Fulvestrant), collagenase type A (from Clostridium histolyticum), 1-chloro-2,4-dinitrobenzene, bovine serum albumin (BSA), trypan blue, L-15 culture medium, dimethyl sulfoxide (DMSO), Triton X-100, sodium dodecyl sulfate, tetramethylethylenediamine, dithiothreitol, ammonium persulfate, urethane, and protease inhibitor cocktail for general use were acquired from Sigma Chemical Co. (St. Louis, MO). 5-Chloromethylfluorescein diacetate (CMFDA) was obtained from Molecular Probes (Eugene, OR). Dulbecco's modified Eagle medium (DMEM) and Williams E medium were from Gibco. 5,6,7,13-Tetrahydro-13-methyl-5-oxo-12H-indolo[2,3-a]pyrrolo[3,4-c]carbazole-12-propanenitrile (Gö6976) was obtained from Calbiochem (San Diego, CA). Rabbit anti-rat sPgp was acquired from Kamiya Biomedical Co. (Seattle, WA). Mouse antihuman MRP2 (M2III-6) was obtained from Alexis Biochemicals (San Diego, CA). Donkey anti-rabbit immunoglobulin G (IgG; 31458), goat anti-mouse IgG (31430), Hyperfilm ECL and Pierce ECL western blotting substrate were obtained from Thermo Fisher Scientific, Inc. (Waltham, MA), anti-ERα, and anti-phosphorylated ERα (pERα; Ser-118) were obtained from Santa Cruz Biotechnologies Inc. (Santa Cruz, CA). Wortmannin (WM) was acquired from Fluka. All other chemicals were of the highest grade available.

### Animals

Adult female Wistar rats weighing 250–300 g and bred in our animal house as described [20], were used in all studies. Treatment were carried out under urethane anesthesia (1 g/kg intraperitoneally), and maintained thus throughout. When necessary, body temperature was measured with a rectal probe and maintained at 37°C. All animals received humane care according to the criteria outlined in the “Guide for the Care and Use of Laboratory Animals” Eighth Edition (National Academy of Sciences, 2011). Experimental procedures were carried out according to the local Guideline for the Use of Laboratory Animals (Resolution N° 6109/012), established by the institutional Bioethical Committee for the Management of Laboratory Animals and approved by the Faculty of Biochemical and Pharmaceutical Sciences of the National University of Rosario.

### Isolation and culture of rat hepatocyte couplets (IRHC)

To obtain a preparation enriched in IRHC, livers were perfused according to the two-step collagenase perfusion procedure and were further enriched by centrifugal elutriation [21], [22]. The final preparation contained 70–80% of IRHC with viability >95%, as assessed by the trypan blue exclusion test. After isolation, IRHC were plated onto 24- well plastic plates at a density of 0.2×10^5^ U/mL in L-15 culture medium, and they were cultured for 5 hours to allow the restoration of couplet polarity [13].

### IRHC treatments

IRHCs were exposed to the vehicle (DMSO; control group) or E17G (25–800 µM) for 20 minutes. To evaluate the role of ERα in the effect of E17G, IRHCs were preincubated with the ERα inhibitor ICI 182,780 (ICI, 1 µM) for 15 minutes, and this was followed by the addition of E17G for another 20-minute period. Studies of ERα and cPKC coinhibition were also carried out by the coadministration of the cPKC inhibitor Gö6976 (1 µM) together with ICI (1 µM) for 15 minutes before exposure to E17G (100 µM) for another 20-minute period. Similarly, studies of ERα and PI3K coinhibition were carried out by the coincubation of IRHC with the PI3K inhibitor Wortmannin (100 nM) together with ICI (1 µM) for 15 minutes before exposure to E17G (100 µM) for another 20-minute period.

### Assessment of Abcb11 and Abcc2 secretory function and localization in IRHC

Transport function of Abcb11 and Abcc2 was evaluated by analyzing the canalicular vacuolar accumulation (cVA) of the fluorescent substrates CGamF and glutathione methylfluorescein (GS-MF), respectively [23], [24]. CGamF is a bile salt analogue transported selectively by Abcb11 [23], whereas CMFDA is a lipophilic compound taken up by passive diffusion across the basolateral membrane and converted into glutathione methylfluorescein (GS-MF) by the sequential action of intracellular esterases and glutathione S-transferases. For transport studies, cells were washed twice with L-15 and exposed to 0.3 µM CGamF [23] or 2.5 µM CMFDA [25], [26] for 15 min. Finally, cells were washed twice with L-15, and canalicular transport activity for both substrates was assessed by fluorescence microscopy [26] under an inverted microscope (Zeiss Axiovert 25). Images were captured with a digital camera (Q-color5 Olympus America Inc., Center Valley, PA), and the cVA of the fluorescent substrates was determined as the percentage of IRHC in the images displaying visible green fluorescence in their canalicular vacuoles from a total analysis of at least 200 couplets per preparation.

To evaluate the intracellular distribution of Abcb11 and Abcc2, IRHC were fixed and stained as previously reported [26]. E17G concentration used in these experiments (200 µM) was higher than that employed in functional experiments to render transporter internalization more evident. The antibodies used were a polyclonal antibody against mouse Abcb11 (anti sPgp, Kamiya Biomedical, Seattle, WA) or a monoclonal antibody against human ABCC2 (M2III-6, Alexis Biochemical, San Diego, CA) (1∶200), followed by incubation with Cy2-conjugated donkey anti-IgG (1∶100) or FITC-labeled goat anti-mouse IgG (1∶100) (Zymed, San Francisco, USA). Cells were then mounted and examined with a Nikon C1 Plus confocal laser scanning microscope, attached to a Nikon TE-2000 inverted microscope. Densitometric analysis of images was made along a line perpendicular to the canalicular vacuole using the Image J 1.44p software (National Institute of Health, USA), as previously described for liver tissue slices [8]. Each measurement was normalized to the sum of all intensities of the respective measurement. The canalicular space was identified on Abcb11/Abcc2-labeled IRHC by superposing each fluorescent image with its respective DIC image [7].

### Western Blot Analysis of ERα Phosphorylation

The activation of ERá was confirmed evaluating the phosphorylation in the Ser-118 amino acid [27], *via* western blotting of the phosphorylated and non-phosphorylated forms of the protein in membrane fractions of hepatocyte primary cultures. Briefly, isolated hepatocytes were obtained by collagenase perfusion [28], and cultured in 3-cm Petri dishes at a density of 2 10^6^ cells/mL. After a 24-h culture period, cells were exposed to E17G (100 µM) for 5 to 20 minutes, then washed with cold 0.3 M sucrose, and finally resuspended in 0.3 M sucrose containing protease inhibitors (Sigma's protease inhibitor cocktail, 1 mM NaF, and 1 mM Na_3_VO_4_), and disrupted *via* sonication. Cytosolic- (supernatant) and total membrane (pellet)-enriched fractions were obtained *via* ultracentrifugation for 60 minutes at 100,000 *g* [29] after elimination of nuclei and cell fragments *via* centrifugation for 10 minutes at 500 *g*. Aliquots containing equivalent total protein content [30] were subjected to sodium dodecyl sulfate/12% polyacrylamide gel electrophoresis. Western blotting in total membrane and cytosolic fractions used an amount of protein that gave a densitometric signal in the linear range for the antibodies used. Separated proteins were electrotransferred to Immobilon-P membranes (Sigma Chemical Co.) and probed with an anti-pSer118 ERá antibody (1∶1000) overnight, after the use of a donkey anti-goat IgG secondary antibody (1∶3000), then membranes were exposed to a chemiluminescence reagent (Pierce ECL), and Hyperfilm ECL. The membranes were then stripped and reprobed with an anti–total ER antibody (1∶1000). pSer118 ERá and total ERá bands were quantified by densitometry with ImageJ 1.44p.

### Western Blot Analysis of PKCc

PKC activation can be estimated by analyzing the translocation of PKC to membrane [31]. Isolated rat hepatocytes were cultured on 3-cm glass Petri dishes at a density of 2 10^6^ cells/mL for 24 hours. Then, cells were exposed to E17G (100 µM) for 5, 10, or 15 minutes, washed with cold 0.3 M sucrose, resuspended in 0.3 M sucrose plus protease inhibitors (inhibitor cocktail, 1 mM NaF, and 1 mM Na_3_VO_4_), and disrupted *via* sonication. In separate experiments, we tested the effect of ICI (1 µM) by exposing the cells for 15 minutes to the inhibitor, prior to adding E17G (100 µM, 5, 10, 15 minutes) or its solvent. ICI was maintained throughout the period of exposure to E17G. Then, cytosolic and total membrane-enriched fractions were obtained by ultracentrifugation as described above. Proteins were separated *via* 10% sodium dodecyl sulfate–polyacrylamide gel electrophoresis; membrane and cytosolic fractions from the same experiment were loaded in the same gel. After the separated proteins were electrotransferred to Immobilon-P membranes and were incubated overnight with monoclonal antibodies against one of the cPKC present in liver, PKCα (human PKCα, BD Biosciences Pharmingen; 1∶1000), followed by incubation with a donkey anti–mouse IgG secondary antibody (1∶3000), membranes were revealed using standard chemiluminescence protocols. Densitometry was performed with ImageJ 1.44p. To estimate the amount of PKCα associated with both cytosolic and membrane fractions, the relative intensity of each band was divided by micrograms of protein loaded in that lane, and then multiplied by the total amount of protein recovered in the corresponding fraction. The proportion of membrane- bound PKCα isoforms was expressed as the amount in membranes (Amembrane) relative to the total cellular amount, according to: Amembrane/(Amembrane + Acytosol).

In separate experiments, we tested the effect of Gö6976 (cPKC inhibitor, 1 µM) on ERα phosphorylation by exposing the cells for 15 minutes to the inhibitor, prior to adding E17G (100 µM, 15 minutes) or its solvent. The Western blot of ERα and p-ERα were performed as described above.

### Synthesis of siRNA

Four 21 nucleotide RNA duplexes (siRNA) targeting rat ERá mRNA were designed using the WIsiRNA selection program [32]. The control siRNA (scrambled) was designed by scrambling the nucleotides of one of these specific targets. The siRNAs were synthesized using the Ambion's Silencer™ siRNA Kit.

### ERα knock-down in sandwich-cultured rat hepatocytes (SCRH)

Hepatocytes were isolated from female Wistar rats as was described previously (28), seeded (9.5×10^5^ cells/well) onto 6-well plates covered with gelled collagen (800 µL of rat tail collagen type I mixed with 100 µL of 0.1 M NaOH and 100 µL of 10× DMEM) and incubated for 2 h at 37°C in Williams E medium with FBS 5% containing antibiotics (gentamicin, streptomycin, penicillin and amphotericin D), dexamethasone 0.8 mg/L, and insulin 4 mg/L. Afterwards, the medium was replaced and cells were incubated for 24 hours before transfection. We optimized transfection of primary hepatocytes by adding 5 µl of lipofectamine (Invitrogen) with 70 nM of siRNA per well, followed by a 4-hour incubation at 37°C.

After transfection, hepatocytes were washed and overlaid with gelled collagen for 1 h at 37°C to obtain a collagen sandwich configuration as was previously described [33]. ERα protein expression was determined by immunoblotting after 48 h of culture in sandwich configuration.

### Assessment of Abcc2 localization in SCRH

To evaluate the intracellular distribution of Abcc2, SCRH were treated with E17G (200 µM, 20 min) or vehicle (DMSO, control) and then fixed with 4% paraformaldehyde in PBS for 30 min, blocked and permeablized with 3% BSA and 0.5% Triton X-100 for 30 min. After that, cells were incubated with a mouse-monoclonal antibody against human ABCC2 (M2III-6, Alexis Biochemical, San Diego, CA) (1∶200) overnight at 4°C, followed by incubation with Cy2-labeled goat anti-mouse IgG (1∶100, 2 h) (Zymed, San Francisco, USA). To delimit the canaliculi, F-actin was stained by coincubating cells with Alexa Fluor 568 phalloidin (Invitrogen, Carlsbad CA) (1∶100, 2 h). Cellular nuclei were stained by incubating during 10 min with 1.5 µM 4,6-diamidino-2-phenylindol (Invitrogen). Finally, cells were mounted and examined with a Nikon C1 Plus confocal laser scanning system, attached to a Nikon TE-2000 inverted microscope. All samples were coded and scored according to morphological criteria (canalicular presence, canalicular length per cell). At least three imaged areas of confluent cells were randomly selected from each culture dish.

### Rat Liver Perfusion

In anesthetized female rats, the bile duct was cannulated with PE-10 tubing (Intramedic, Clay Adams). Livers were perfused *in situ via* the portal vein in a non-recirculating single-pass design with Krebs-Ringer bicarbonate at 37°C, equilibrated with 5% CO_2_/95% O_2_, at a constant flow rate of 30 mL/min. Taurocholate (2.5 µmol/L) and 1-chloro-2,4-dinitrobenzene (0.5 µmol/L) were added to the perfusion medium for bile salt and dinitrophenyl-glutathione (DNP-G) secretion studies. After a 20-minute equilibration period, the inhibitor ICI (0.5 µM final concentration) or its solvent (DMSO, 370 µL/L) was added to the reservoir. Fifteen minutes later, a 5-minute basal bile sample was collected, followed by administration of E17G (3 µmol/liver, intraportal single injection over a 1-minute period) or its solvent (DMSO/10% BSA in saline [4∶96]), and bile collected at 5-minute intervals for an additional 60 minute. Experiments were considered valid only if initial bile flow (after equilibration) was greater than 30 µL/min/kg. Viability of the liver was monitored *via* lactate dehydrogenase activity in the perfusate outflow; experiments exhibiting activities over 20 U/L were considered invalid. Transport activities of Abcc2 and Abcb11 were evaluated by measuring biliary DNP-glutathione and taurocholate excretion, respectively. DNP-G content was assessed in all samples by high-performance liquid chromatography, as described previously [34], using authentic standards. Total bile salt concentration was assessed using the 3α-hydroxysteroid dehydrogenase procedure [35] and the result was assumed as TC concentration.

For canalicular transporter localization studies, in a new set of experiments, a liver lobe was excised 10 minutes after the addition of E17G, frozen immediately in isopentane precooled in liquid nitrogen, and stored at −70°C for further immunofluorescence and confocal microscopy analysis. Liver sections were obtained with a Zeiss Microm HM500 microtome cryostat, air-dried, and fixed with 3% paraformaldehyde in phosphate-buffered saline for colocalization studies. After fixation, liver slices were incubated overnight with the specific antibodies to Abcb11, Abcc2, and occludin, and this was followed by 1 hour of incubation with the appropriate cyanine 2-conjugated or cyanine 3-conjugated donkey anti-IgG. Occludin staining was carried out to demarcate the limits of the canaliculi [7], [36]. All images were taken with a Nikon C1 Plus confocal laser scanning microscope. To ensure comparable staining and image capture performance for the different groups belonging to the same experimental protocol, liver slices were prepared on the same day, mounted on the same glass slide, and subjected to the staining procedure and confocal microscopy analysis simultaneously. Image analyses of the degree of Abcb11 and Abcc2 endocytic internalization were performed on confocal images with ImageJ 1.44p (National Institutes of Health), as described elsewhere [7].

### Western Blot Analysis of ERα Phosphorylation *in Vivo*

Anesthetized female rats received E17G (15 µmol/kg) or solvent through the femoral vein. Immediately after, partial hepatectomies were performed at different times (5, 10, 15, 20 min). Liver samples were homogenized in sucrose 0.3 M containing protease inhibitors (Sigma's protease inhibitor cocktail, 1 mM NaF, and 1 mM Na_3_VO_4_), and disrupted *via* sonication. The activation of ERα was confirmed by an evaluation of the phosphorylation in the Ser118, *via* western blotting in homogenates of liver as indicated above.

### Statistical analysis

Results are expressed as mean ± standard error of the media (SEM). One-way ANOVA, followed by Newman-Keuls' test, was used for multiple comparisons. The variances of the densitometric profiles of Abcb11 and Abcc2 localization were compared with the Mann-Whitney U test. The four-parameter dose-response curves were compared using GraphPad Prism software (GraphPad Software Inc., La Jolla, CA). Values of p<0.05 were considered to be statistically significant.

## Results

### E17G Activates ERα

**Figure 1:**
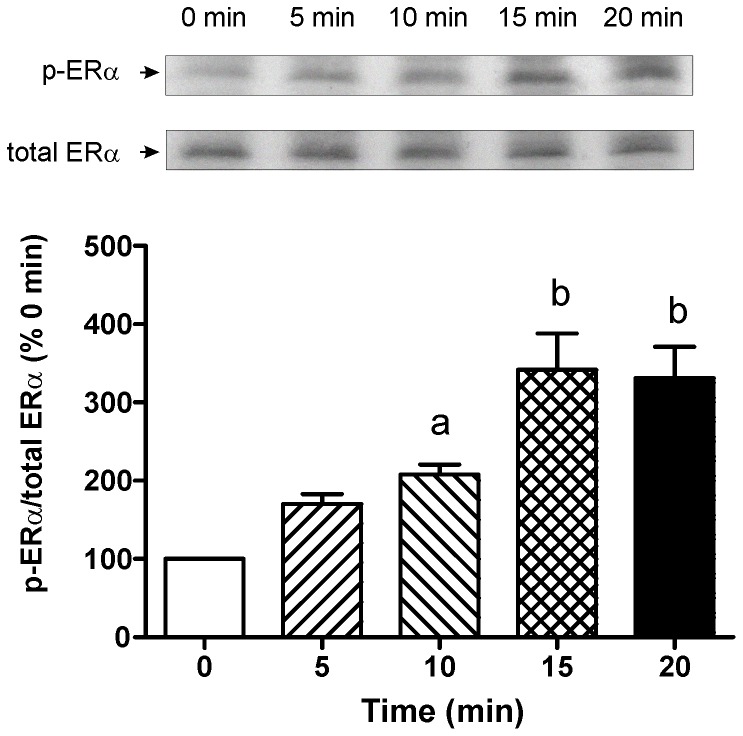
Estimation of Estrogen Receptor α (ERα) activation by estradiol 17ß-d-glucuronide (E17G). Isolated rat hepatocytes were incubated with **E17G** (100 µM) during different time-periods (0 to 20 min). ERα activity was determined by immunoblotting using antibodies against phosphorylated ERα (p-ERα Ser118) and total ERα. Phosphorylation degree of ERα was calculated as the ratio of each p-ERα total ERα band intensity and expressed as percent of this ratio at 0 min of E17G exposure.. Data are expressed as mean ± SEM (n = 3). ^a^ Significantly different from 0 min (p<0.05). ^b^ Significantly different from 0 min and 10 min of E17G treatment (p<0.05).

Western blots of p-ERα (Ser118), an indicator of ERα activation, showed that E17G increased the amount of p-ERα in a time-dependent manner in membrane fractions (Fig. 1). This increase reached a peak at 15 minutes and was verified in cell homogenates and in cytosolic ER (data not shown).

### ICI 182,780 partially prevented E17G-induced impairment of canalicular secretory function

To assess which concentration of ICI produced the maximal protective effect, we performed concentration–response studies varying the inhibitor concentration (0.01–10 µM) with a fixed E17G concentration (100 µM). ICI partially prevented the effect of E17G on canalicular vacuolar accumulation (cVA) of cholyl-glycylamido-fluorescein (CGamF) and glutathione methylfluorescein (GS-MF) throughout the range of concentrations evaluated except 10 µM ICI preventive effect was maximal at the concentration of 1 µM, hence the remaining experiments in IRHC were performed using these concentrations. ICI alone did not modify cVA of CGamF and GS-MF respect to control (Figure S1) and did not alter neither cell morphology nor cell viability (data not shown).

**Figure 2:**
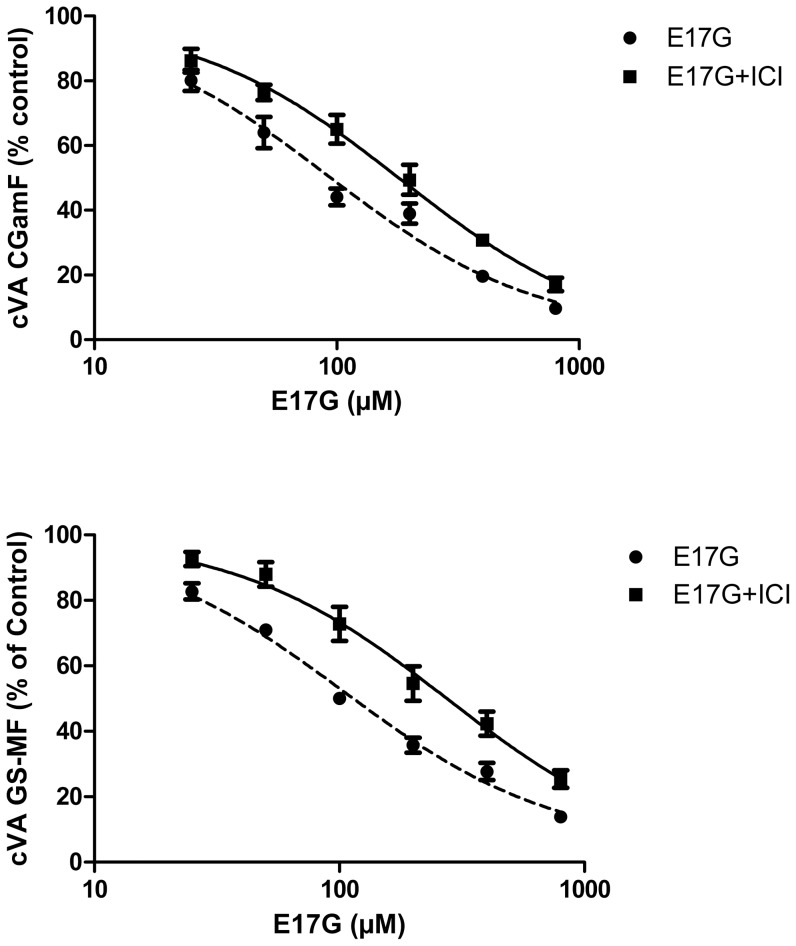
Prevention by ICI 182,780 (ICI) of estradiol 17ß-d-glucuronide (E17G)-induced impairment of canalicular vacuolar accumulation (cVA) of CGamF (upper panels) and GS-MF (lower panels). IRHC were preincubated with ICI (1 µM) for 15 minutes, and then exposed to E17G (25–800 µM) for an additional 20-min period. cVAs of CGamF and GS-MF were calculated as the percentage of couplets displaying visible fluorescence in their canalicular vacuoles from a total of at least 200 couplets per preparation, referred to control cVA values. cVA control values were: 66±3% for CGamF and 76±3% for GS-MF and cVA of IRHC treated with ICI alone were 65±2% for CGamF and 76±4% for GS-MF. Data are expressed as mean ± SEM (n = 3).

To characterize the protective effect of ICI, we carried out different concentration–response studies using variable concentrations of E17G and a fixed concentration of the inhibitor. Curves were adjusted assuming that the parameter minimal effect (bottom) was equal to 100% (similar to control) and that the Hill slope coefficient was 1. ICI (1 µM) significantly prevented the E17G-induced decreases in cVA of CGamF and GS-MF in all the range of E17G concentrations tested (Fig. 2). The IC50 of CGamF and GS-MF accumulation induced by E17G (91±1 µM and 104±1 µM, respectively) was significantly increased in the presence of ICI by 102 and 164%, respectively (E17G+ICI, IC50 for CGamF: 184±1 µM, for GS-MF: 275±2, p<0.05).

### ICI 182,780 prevented E17G-induced internalization of canalicular transporters Abcb11 and Abcc2

**Figure 3:**
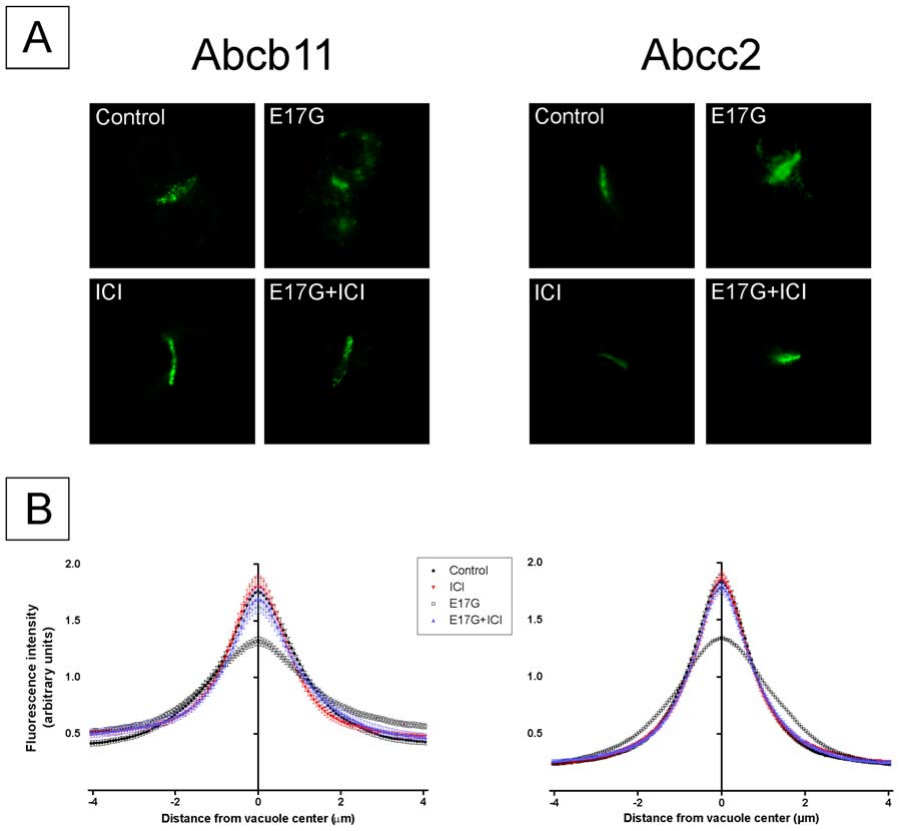
Estrogen receptor inhibitor ICI 182,780 (ICI) prevented estradiol 17ß-d-glucuronide (E17G)-induced endocytic internalization of Abcb11 and Abcc2 in IRHC. Panel A shows representative confocal images depicting cellular distribution of the canalicular transporters studied under the different treatments. Note that under control or ICI conditions transporter-associated fluorescence is mainly localized at the canalicular membrane. E17G (200 µM) induced a clear internalization of transporter-containing vesicles beyond the limits of the canaliculus, phenomenon significantly prevented by pretreatment with ICI (1 µM, 15 min). Panel B shows the densitometric analysis of the distribution of Abcb11 and Abcc2 fluorescence intensity along an 8-µm line perpendicular to the canalicular vacuole (4 µm to each side of the vacuole center), using the ImageJ 1.44p software (NIH, USA). The canalicular space was identified based on the corresponding DIC image. Each line profile measurement was normalized to the sum of all intensities of the respective measurement. The distribution of Abcb11 or Abcc2 fluorescence, expressed as a percentage of the total, was then calculated for each canaliculus and compared statistically, using the Mann-Whitney test. Statistical analysis of the profiles revealed a significant internalization of Abcb11 and Abcc2 under E17G treatment (p<0.05 vs control), which was completely abolished by ICI (p<0.05 vs E17G). Results are expressed as mean ± SEM. n = 6–8 canalicular vacuoles per preparation, from 3 independent preparations.

The effect of E17G on Abcb11 and Abcc2 function was accompanied by a significant redistribution of these transporters from the canalicular membrane into intracellular vesicles (Fig. 3 A). The pretreatment of IRHCs with ICI markedly prevented this delocalization. This was confirmed by densitometric analysis, which demonstrated an E17G-induced redistribution of both Abcb11 and Abcc2 over a greater distance from the canalicular vacuoles that was fully prevented by ER blockage (Fig. 3 B).

### ERα acts complementarily with PI3K but not with cPKC in the E17G-Induced Canalicular Secretory Failure

**Figure 4:**
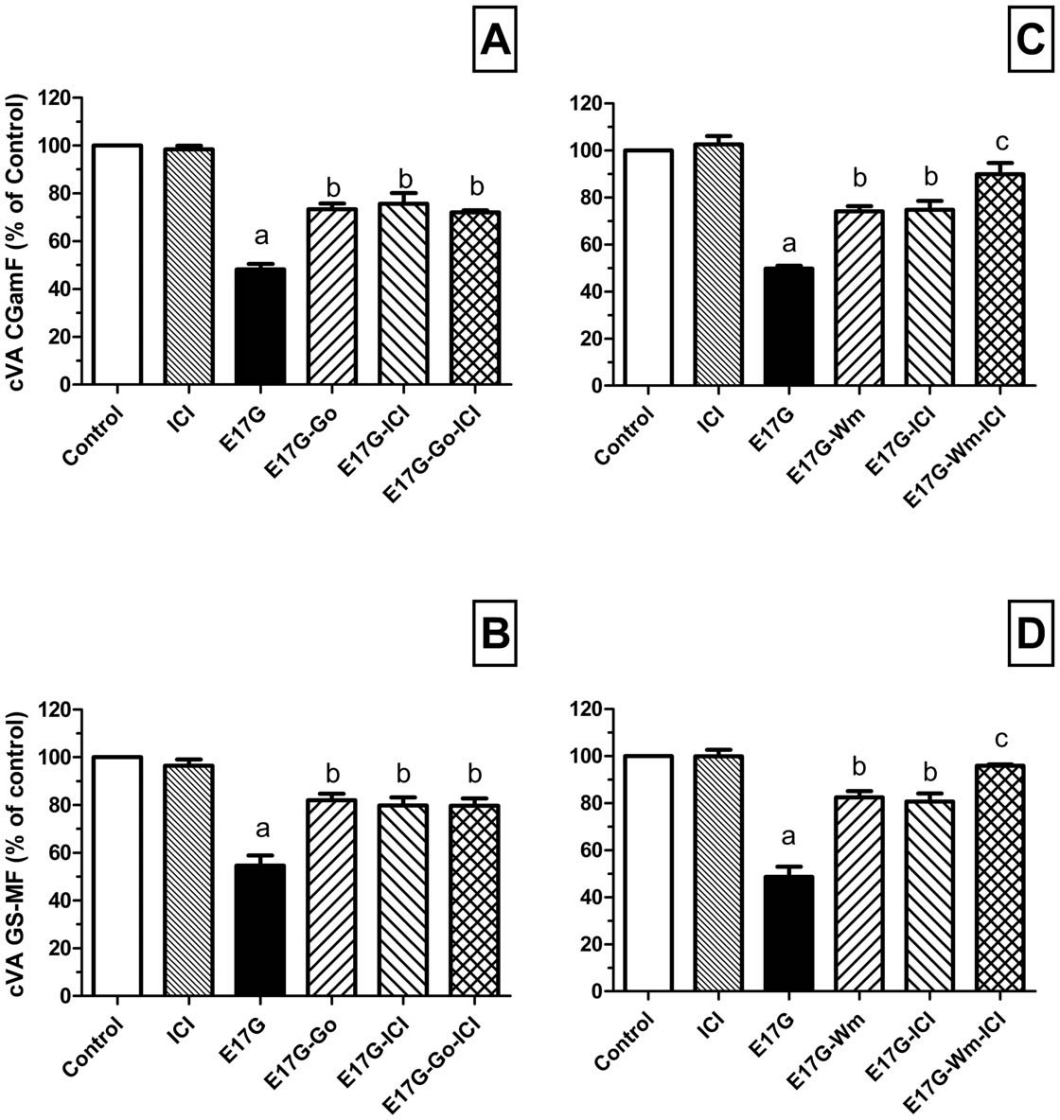
Effect of co-incubation with ICI 182,780 (ICI) and classic protein-kinase C (cPKC) or phosphoinositide 3-kinase (PI3K) inhibitors on estradiol 17ß-d-glucuronide (E17G)-induced impairment of canalicular vacuolar accumulation (cVA) of CGamF (panels A and C) and GS-MF (panels B and D). Left panels: IRHC were incubated with Gö6976 (Gö, cPKC inhibitor, 1 µM) and ICI (1 µM), either alone or together, for 15 min. Right panels: IRHC were incubated with wortmannin (Wm, PI3K inhibitor, 100 nM) and ICI (1 µM), either alone or together, for 15 min. Then, IRHC were exposed to E17G (100 µM) for an additional 20-min period. Finally cVAs of CGamF and GS-MF were calculated as the percentage of couplets displaying visible fluorescence in their canalicular vacuoles from a total of at least 200 couplets per preparation, referred to control cVA values. Control cVA values were:67±3% for CGamF and 75±1% for GS-MF. Data are expressed as mean ± SEM (n = 3). ^a^ Significantly different from control (p<0.05). ^b^ Significantly different from E17G and control (p<0.05). ^c^ Significantly different from E17G, E17G+Wm and E17G+ICI (p<0.05).

The preventive effects of ICI (1 µM) and WM (100 nM) on the decreases in cVA of CLF and GS-MF induced by E17G were additive (Fig. 4, C and D), suggesting that ERα and PI3K act in different pathways. Contrarily, no additive effect was observed with ICI (1 µM) and Gö6976 (1 µM) (Fig. 4, A and B), hence it is possible that ERα and cPKC share a common pathway and this finding deserved further studies to analyze the sequence of activation. It is worth noting that the concentration of the inhibitors employed produced the maximal protective effects allowing us to speculate about additive effects.

### The Activation of ERα precedes that of cPKC

**Figure 5:**
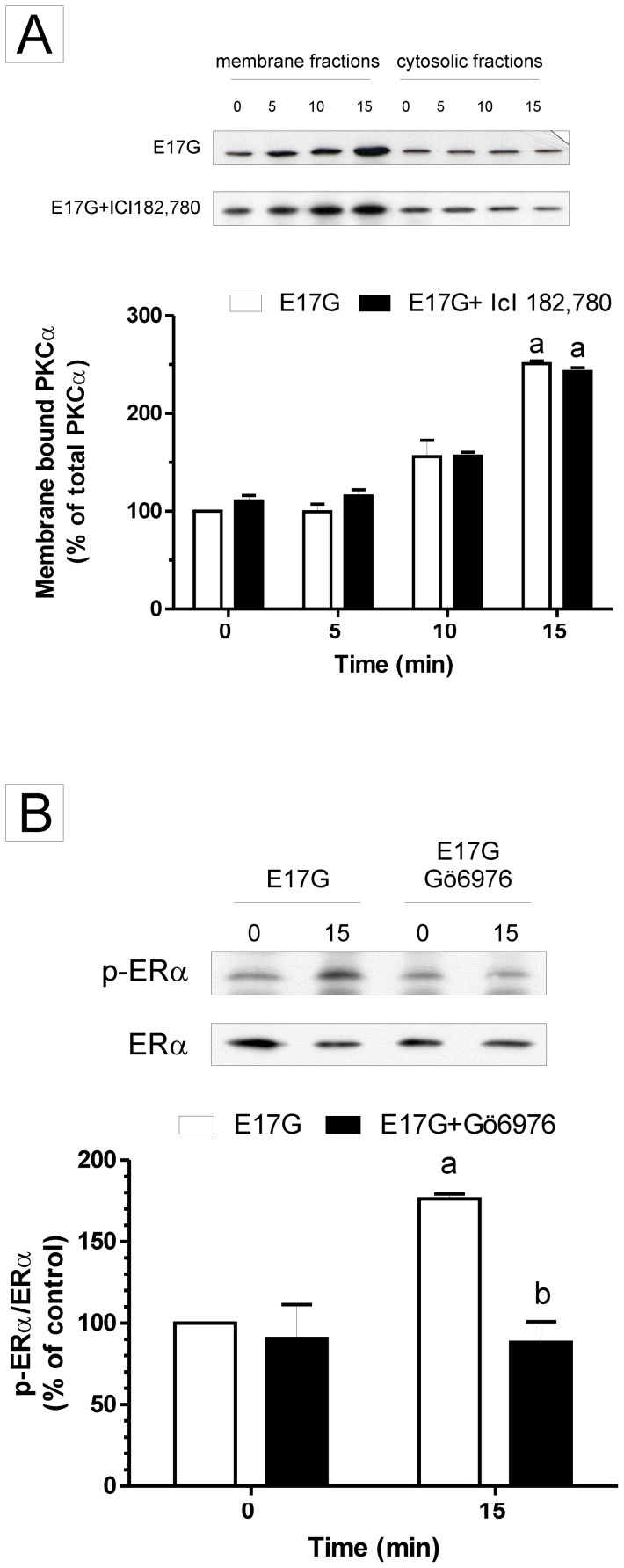
Activation of estrogen receptor α (ER α) and classic protein-kinase C α (PKCα) in the presence of the cross inhibitors Gö6976 (Gö) and ICI 182,780 (ICI), respectively. Panel A. Evaluation by immunoblotting of the effect of ICI on the specific PKCα activation by E17G in primary cultured hepatocytes. Primary cultured hepatocytes were treated with ICI (1 µM) for 15 min, then exposed to E17G (100 µM) for 5, 10 and 15 minutes, and finally the distribution of PKCα between cytosol and membrane was evaluated. The bar graph shows the fold translocation of PKCα isoform. PKCα at time 0 min without ICI (Control cells) were considered to be 1.0-fold activated. Area under the peak of the PKC isoform scanned (both cytosolic and membrane fractions) was determined, and the membrane-to-cytosol ratio was used to calculate fold translocation (or activation). Panel B. Effect of Gö on ERα activation by E17G. Isolated rat hepatocytes were incubated with Gö (1 µM) for 15 min and the exposed to E17G (100 µM) for another 15 min-period. ERα activity was determined by immunoblots using antibodies against phosphorylated ERα (p-ERα, Ser118) and ERα. The ratio of each p- ERα/ERα band density was compared to control bands ratio (100%). Data are expressed as mean ± SEM; n = 3 Western blot analyses, each from different cell culture experiments. ^a^ Significantly different from control (p<0.05). ^b^ Significantly different from E17G (p<0.05).

To confirm the temporal activation of ERα and cPKC, two different experiments were performed. First, Fig. 5 (panel A) shows that pretreatment with ICI did not prevent the activation of PKCα induced by E17G observed through membrane translocation as a measure of activation (by Western blot) discarding that ER activation was previous to that of cPKC. Secondly, panel B of Fig. 5 indicates that pretreatment with Gö6976 prevented the activation of ERα induced for E17G indicating that the cPKC activation occurs before that ERα.

### Effect of ERα knock-down on estradiol 17ß-d-glucuronide (E17G)-induced endocytic internalization of Abcc2

**Figure 6:**
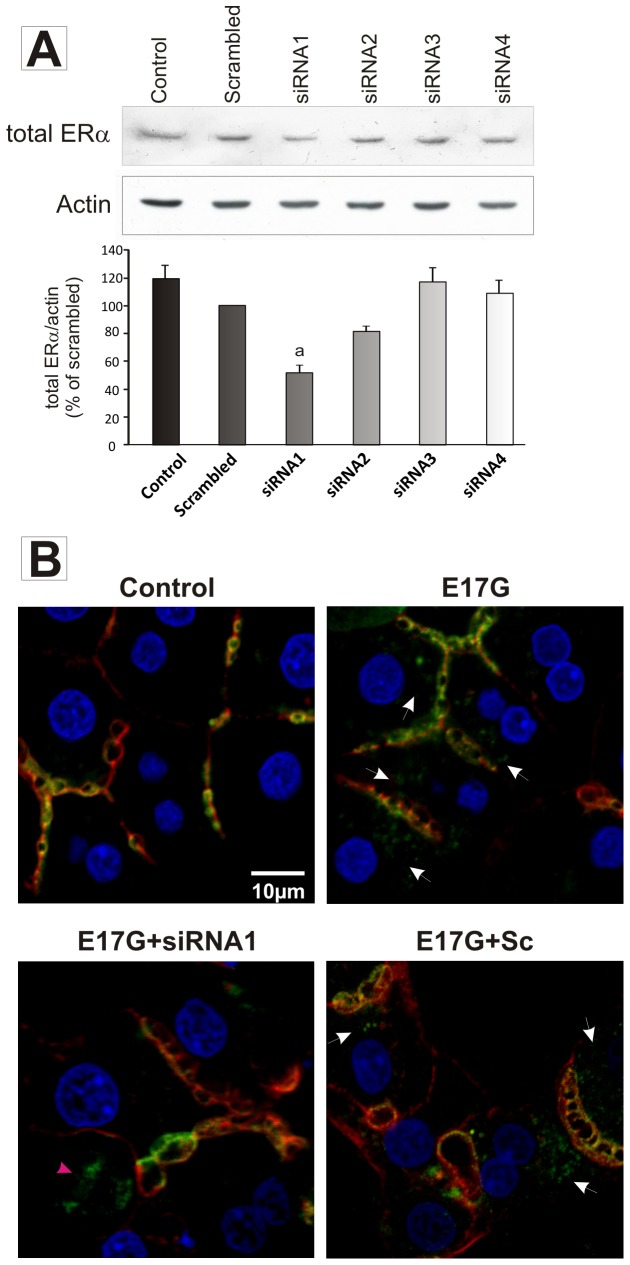
Estrogen receptor α (ERα) knock-down prevents estradiol-17ß-d-glucuronide (E17G)-induced endocytic internalization of Abcc2 in sandwich-cultured rat hepatocytes (SCRH). Panel A: Representative western blot of ERα in SCRH transfected with four different siRNA. The siRNA1 induced a significant decrease in ERα expression (51±3% of scrambled siRNA-treated SCRH, p<0.05). ^a^ significantly different from scrambled. Panel B: Representative confocal images showing cellular distribution of Abcc2 (green) in SCRH. Actin network (red) and nuclei (blue) are also shown. E17G induced a clear internalization of Abcc2, visualized as transporter-containing vesicles beyond the canalicular region, reaching the perinuclear zone (white arrowheads). In cells treated with siRNA1 this phenomenon was significantly preventive only in cells effectively transfected. Cells that were not transfected showed the typical pattern of Abcc2 delocalization (pink arrowheads). Scrambled-transfected cells also showed a pattern of Abcc2 delocalization after E17G treatment.

To confirm the participation of ERα in E17G-induced cholestatic alteration, we evaluated the localization status of Abcc2 in SCRH transfected with siRNA targeting rat ERá mRNA. Four different siRNAs were tested and the siRNA1, targeting rat ERα nucleotides 898–916 (CCAATGCACCATCGATAAG) induced a significant decrease in ERα expression, as analyzed by immunoblotting (see Fig. 6A) and was chosen for transporter localization studies.

Figure 6B shows representative confocal images for Abcc2 where it can be appreciated that E17G induced redistribution of Abcc2 to the inner part of the cells. ERα knockdown prevented the effect of E17G on Abcc2 localization giving additional support to a role of ERα in the pathway that leads to E17G-induced internalization of canalicular transporters and the resulting secretory failure. Cells treated with scrambled siRNA showed the same delocalization pattern that E17G.

### ERα activation *in vivo*

**Figure 7:**
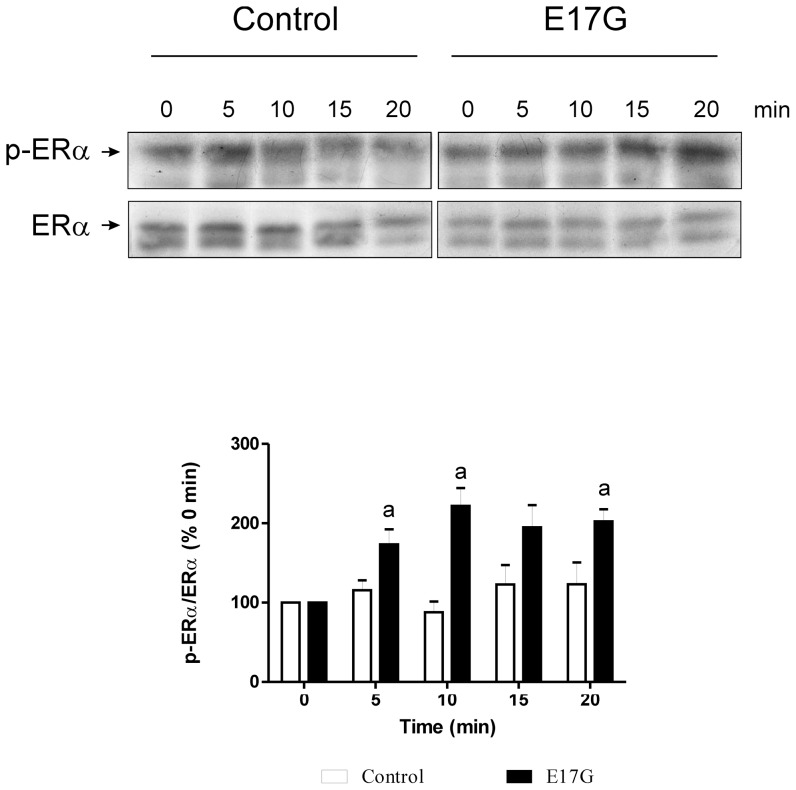
Estrogen Receptor α (ERα) activation by estradiol-17ß-d-glucuronide (E17G) *in vivo*. E17G (15 µmol/kg) or solvent was administered through the femoral vein. Immediately after E17G administration, partial hepatectomies were performed at different times (5, 10, 15, 20 min). ERα activity was determined by immunoblots using antibodies against phosphorylated ERα (p-ERα, Ser118) and ERα. The ratio of each p-ERα/ERα band density was compared to bands ratio of time 0 min (100%). Data are expressed as mean ± SEM (n = 3). ^a^ Significantly different from control sample at the corresponding time (p<0.05).

*In vivo*, E17G also activated ERα. In liver homogenates, activation was maximal 10 min after the injection of the estrogen and ERα remained activated 20 min after E17G injection (Fig. 7). Activation of membrane associated ERα was not significant although there was a trend (data not shown).

### ERα Is Involved in the Decay of Bile Secretory Function Induced by E17G in the PRL Model

**Figure 8:**
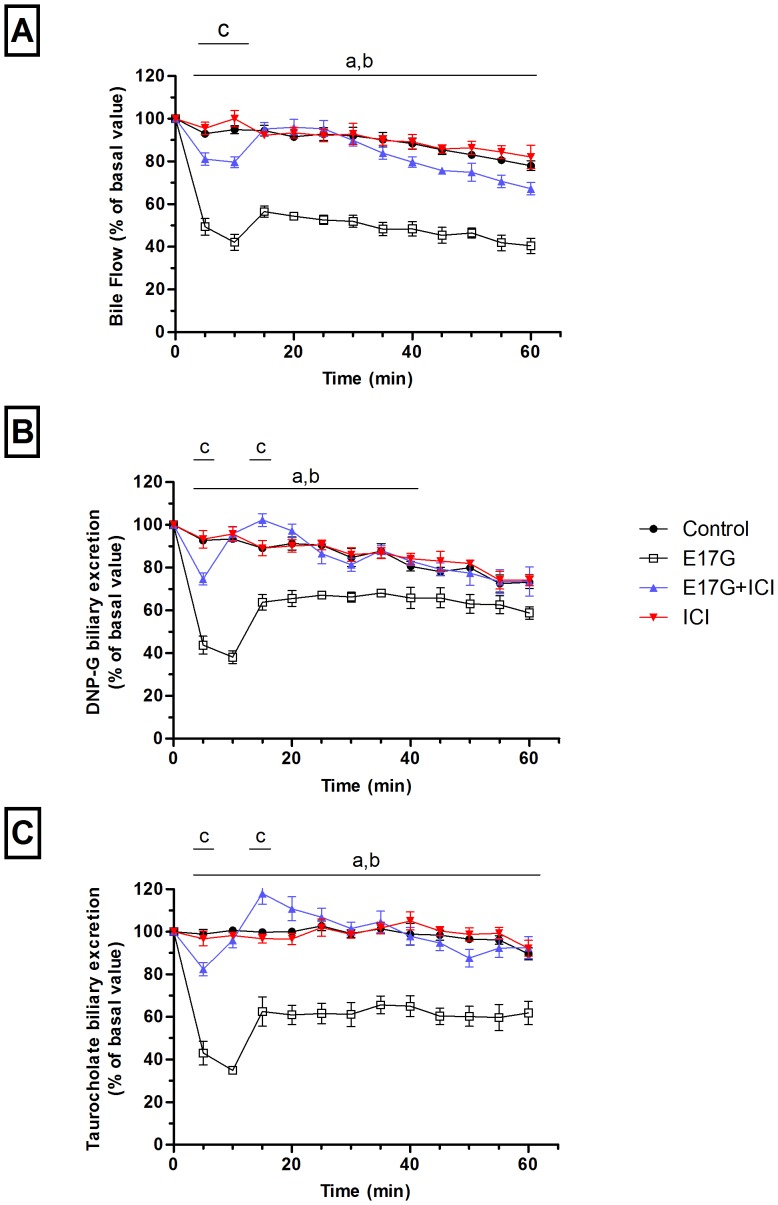
ICI 182,780 (ICI) protects against estradiol-17ß-d-glucuronide (E17G)-induced impairment of bile flow and biliary secretion of dinitrophenyl-glutathione and taurocholate in the perfused rat liver. Temporal changes in bile flow (panel A) and in the biliary excretion rate of both total dinitrophenyl-glutathione (DNP-G, panel B) and taurocholate (panel C) throughout the perfusion period. PRLs were treated with a bolus of E17G (3 µmol/liver) or with the E17G vehicle DMSO/BSA 10% in saline (control), in the presence or absence of ICI (0.5 µM). ^a^ Control significantly different from E17G, ^b^ Control significantly different from E17G+ICI, ^c^ E17G significantly different from E17G+ICI. (p<0.05). N = 3–4 animals per group.

The acute, initial reduction in bile flow due to transporter endocytosis after E17G administration and the subsequent recovery due to reinsertion of these transporters occur differentially in time. They can therefore be readily dissected with the PRL model, which allows dynamic monitoring of changes in bile secretory function. The bolus administration of E17G decreased bile flow to a minimum of approximately 40% of basal flow within 10 minutes, and the bile flow did not recover throughout the perfusion period (Fig. 8, panel A). This was accompanied by a decrease in the biliary excretion of the Abcc2 and Abcb11 substrates DNP-glutathione (minimum 40%) and taurocholate (minimum 35%), respectively. Both transport activities recovered to approximately 60% of basal values from 15 min after E17G administration onwards (Fig. 8, panels B and C). The cumulative excretion of DNP-G decreased to 72±2% of control excretion, whereas TC cumulative excretion was 59±4% of control after E17G administration. ICI prevented the initial drop in bile flow (minimum 78%) and completely prevented bile flow alterations induced by E17G from 15 min onwards. ICI preadministration also preserved the biliary excretion of Abcc2 and Abcb11 substrates in E17G-treated rats where DNP-G excretion reached a minimum of 75% and that of TC reached a minimum of 82%. Then, both substrates excretion in ICI+E17G rats increased and overtook substrate excretion in control rats 15 min after E17G (or DMSO) injection, being similar to control afterwards. As a result, the cumulative excretion of DNP-G (99±2% of control) and TC (99±3% of control) in ICI+E17G rats did not differ from that of control rats.

**Figure 9:**
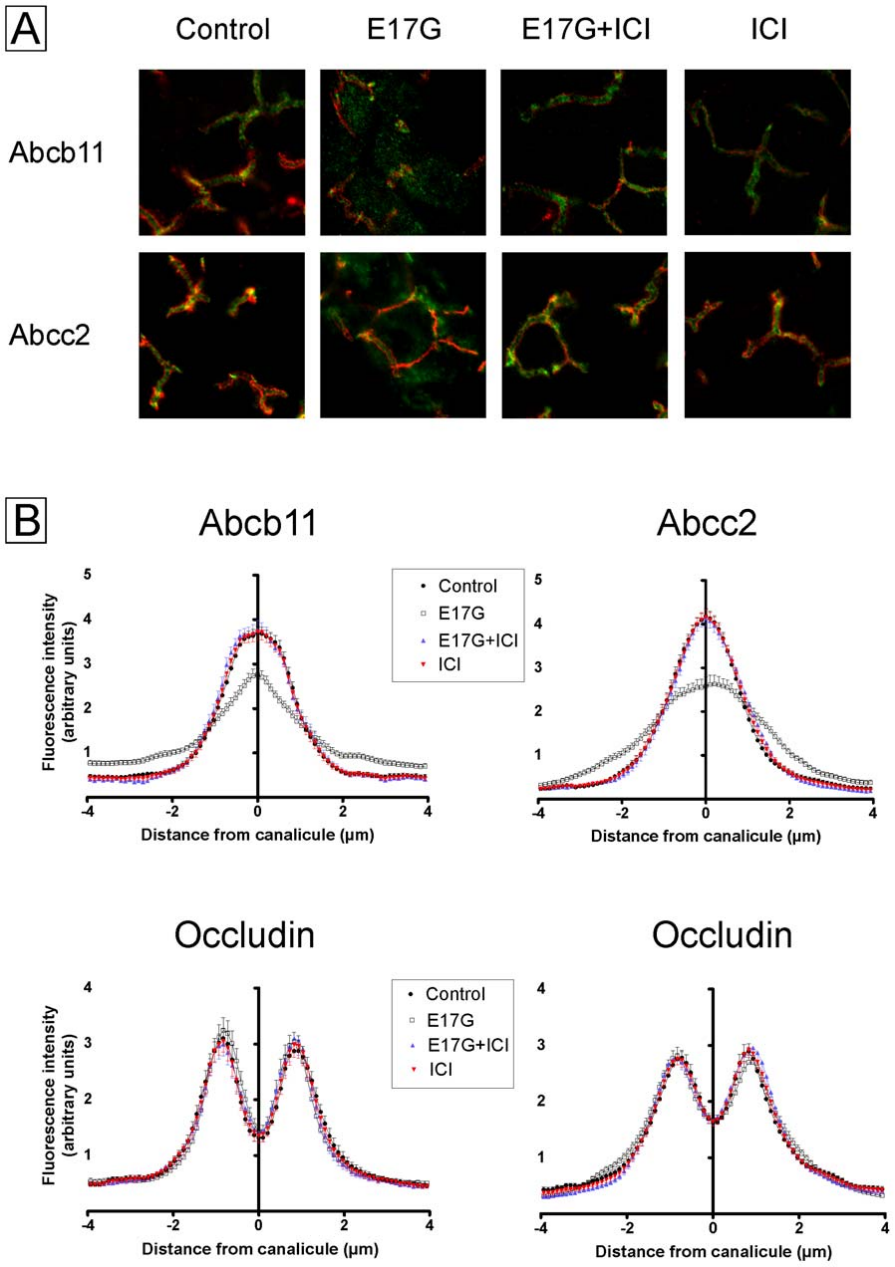
Estrogen Receptor inhibition prevents estradiol-17ß-d-glucuronide (E17G)-induced endocytic internalization of Abcb11 and Abcc2 in perfused rat liver. Panel A: Confocal images of E17G-induced internalization of Abcb11 and Abcc2 and protection by ICI 182,780 (ICI). Representative confocal images of immunostained liver samples displaying a containing of Abcb11 (green) and occludin (red) (upper images), and Abcc2 (red) and occludin (green) (lower images). In control livers, both Abcb11 and Abcc2 were mainly confined to the canalicular space delineated by the tight junction-associated protein occludin. Following E17G (3 µmol/liver), some canaliculi show intracellular fluorescence associated with Abcb11 or Abcc2 at a greater distance from the canalicular membrane, consistent with their delocalization. ICI (0.5 µM, 15 min previous to E17G) prevented the internalization of canalicular transporters, as illustrated by a control-like pattern of Abcb11 and Abcc2 distribution. ICI itself did not induce any changes in transporters localization. Panel B: Densitometric analysis of fluorescence intensity profile of Abcb11, Abcc2 and occluding. Graphs represent the intensity of fluorescence associated with the transporters along an 8-µm line (from −4 µm to +4 µm of the canalicular center) perpendicular to the canaliculus. In control livers, transporter-associated fluorescence was concentrated in the canalicular space. E17G-induced internalization of transporters from the canalicular membrane (P<0.01 versus control) was detected as a decrease in the fluorescence intensity in the canalicular area together with an increased fluorescence at a greater distance from the canaliculus. Distribution profiles of livers treated with E17G+ICI was similar to control and indicated a significantly decreased of Abcb11 and Abcc2 internalization (P<0.01 versus E17G). (n = 20–50 canaliculi per preparation, three independent preparations). Statistical analysis of the distribution profiles of occluding, used to demarcate limits of the canaliculi, showed no changes in the normal distribution by any of the treatments.

Fig. 9 A shows confocal images of Abcb11 (green) and occludin (red) (upper images), or Abcc2 (green) and occludin (red) (lower images) 10 minutes after E17G (or DMSO) administration. In E17G-treated livers, both Abcb11 and Abcc2 were detected in intracellular structures, consistent with their endocytic internalization from the canalicular membrane. This pattern of internalization was evident in some canalicular structures and coexisted with preserved canalicular localization of the transporters at other sites. In densitometric studies, E17G-treated livers showed a wider and flatter profile, consistent with increased fluorescence at a greater distance from the canalicular membrane, indicative of internalization of these transporters into the intracellular compartment (Fig. 9 panel B). In livers perfused with E17G + ICI, the distribution of both Abcb11 and Abcc2 was almost identical to that in control livers and this was confirmed by densitometric analysis. Neither E17G nor ICI altered the canaliculus width estimated by the two densitometric peaks of occludin. ICI itself did not induce any changes in the distribution of Abcc2 and Abcb11 (Fig. 9 B).

## Discussion

Within the last two decades, several pieces of evidence have been provided to prove the involvement of intracellular signaling cascades in onset of the cholestatic phenomena [10]–[12], [14], [37], [38]. Our group has recently demonstrated that cPKC accounts in part for the acute cholestasis caused by E17G, whereas novel PKC like PKCε did not participate in the pathology [12]. Activation of cPKC induced by this cholestatic agent correlates well with its ability to induce endocytic internalization of canalicular transporters critical for bile secretion, such as Abcc2 and Abcb11 [12]. The actions of cPKC in the acute cholestasis caused by E17G do not account, however, for all of the phenomena observed, and other signaling pathways may be involved. A likely candidate is the PI3K-dependent transduction pathway. Supporting this hypothesis, Boaglio et al. [13] showed for the first time that E17G activates PI3K in the liver, and that this event is involved in the cholestatic effects of the estrogen.

Several studies have demonstrated that non-conjugated estradiol is able to activate cPKC and PI3K in different tissues, including liver [15], [16]. These activations are in some cases dependent on the activation of ER [15]. This receptor is central in both non-genomic and genomic effects of estradiol. No evidence existed about the participation of ER in estradiol-17ß-D-glucuronide induced cholestasis and this manuscript gives a first insight in the role of this receptor in the alteration of canalicular transporter function. There are two variants of ER, ERα y ERß being the former the only one expressed in the hepatocyte [18].

E17G activated ERα, similarly to what was observed with estradiol [27]. In PRL, E17G activation was significant 5 min after estrogen administration, which is coincident with the decrease in transport activities and previous to the maximal decrease in transport induced by E17G. The participation of ER activation in the development of estrogen cholestasis was confirmed with the use of ICI 182,780 also known as fulvestrant, an ER inhibitor with no estrogen activity [39] approved for the treatment of hormone receptor-positive metastatic breast cancer in postmenopausal women [40]. This inhibitor partially prevented the decrease in Abcb11 and Abcc2 activity induced by E17G and protected transporter delocalization induced by the estrogen as evaluated with confocal microscopy. Participation of ERα in E17G-induced delocalization of Abcc2 was confirmed using ERα knock-down hepatocytes. The role of ERα in the effects of E17G on transport activity and transport delocalization was not only demonstrated *in vitro* but also in a more complex and physiologic model as the PRL. The role of ER in E17G cholestasis was also more evident in the latter model where ICI prevented almost completely the effect of E17G on bile flow and on the excretion of Abcb11 and Abcc2 substrates, TC and DNP-G. Although there was a slight but significant decrease in these parameters 10 min after E17G administration in ICI-treated rats, immediately there was a rebound excretion and the total excretion of TC and DNP-G were similar to control rats.

Given the fact that Boaglio et al [13] described that PI3K and cPKC were complementary in their participation in E17G cholestasis since the inhibition of both proteins showed an additive effect to protect from the alteration in canalicular transport, we were interested in knowing with which pathway ER was associated, if any. PI3K and ER coinhibition was additive suggesting that they belong to different pathways, whereas cPKC and ER coinhibition was not additive suggesting that they share a common pathway. Next, we focused in the relationship between cPKC and ER. Through the combination of the inhibition of one protein and the measurement of the other protein activation by western blot, the experiments demonstrated that cPKC activation preceded that of ER. These findings do not necessarily indicate that PKC phosphorylates ER since it is possible the existence of other intermediate signaling molecules. GSK-3ß has been proposed as the enzyme that phosphorylates ER in ser118 [41] and Goode et al [42] demonstrate that cPKC could phosphorylate GSK-3ß. Other possible intermediate target of cPKC could be MAPK [43] which also is able to phosphorylate ER in ser118 [44].

Considering that according to the data presented, ER activation by E17G requires the previous activation of PKC, the question is which is the initial receptor of E17G. One possible mechanism is that E17G binds first to ER, then the complex E17G-ER activates PKC and finally PKC *per se* or *via* an intermediate kinase phosphorylates ER. Other, more probable receptor is GPR30, another estrogen receptor located in plasma membrane and in the endoplasmic reticulum that has been recently implicated in E17G-induced cholestasis [45]. Finally, it is not possible to discard that PKC itself acts as the initial binding partner of E17G, leading to its activation and the further phosphorylation of ER [46].

How ERα activation leads to transporter endocytic desinsertion cannot be deduced from our data. ER can trigger nuclear and extranuclear pathways. The role of genomic effects cannot be ruled out, however, the period of time required to protein synthesis seems to exceed the time employed by E17G to exert its cholestatic effects. Extranuclear effects seem more probable. Membrane-bound ER is known to interact with membrane and cytoplasmic adaptor proteins including caveolins, striatin, p130Cas, Shc, HPIP, MTA-1s, and MNAR/PELP1 [47]. Through the interaction with these proteins, ER activates signaling pathways such as Src/MAPK and PI-3 kinase/Akt. This latter is unlikely by our evidence that indicates that ER and PI3K would participate in different pathways; however, MAPK is a likely effector to continue the cascade of protein activation leading to transporter endocytic internalization.

In conclusion, this study demonstrates the participation of ER in E17G-induced cholestasis. This receptor is activated by E17G and this activation is necessary for the desinsertion of the canalicular transporters Abcc2 and Abcb11. We also placed this protein in one of the two signaling pathways that leads to estrogen cholestasis described so far. E17G first activates cPKC and then this protein *per se* or probably by other intermediate signaling proteins activates ER that finally triggers transporter desinsertion through the activation of yet unknown mediators. The pathway that involves PI3K, allegedly responsible of maintaining canalicular transporters desinserted would be independent of ER activation in E17G cholestasis.

## Supporting Information

Figure S1 **Determination of maximal prevention by ICI 182,780 (ICI) against E17G-induced impairment of canalicular vacuolar accumulation (cVA) of CGamF (left panel) and GS-MF (right panel).** IRHC were preincubated with ICI (0.01–10 µM) for 15 minutes, and then exposed to E17G (100 µM) or DMSO (Control) for an additional 20-min period. cVAs of CGamF and GS-MF were calculated as the percentage of couplets displaying visible fluorescence in their canalicular vacuoles from a total of at least 200 couplets per preparation, and expressed as percentage of control cVA values. Dotted line represents the cVA of IRHC exposed to E17G (100 µM) alone. ICI itself did not induce any changes in cVA of CGamF and GS-MF. Data are expressed as mean ± SEM (n = 3).
(ZIP)
