# Supplementary figures and images for: Sequential Activation of Classic PKC and Estrogen Receptor α Is Involved in Estradiol 17ß-D-Glucuronide-Induced Cholestasis
Source: PLoS One. 2012 Nov 27;7(11):e50711. doi: 10.1371/journal.pone.0050711 (PMC3507741; doi:10.1371/journal.pone.0050711)

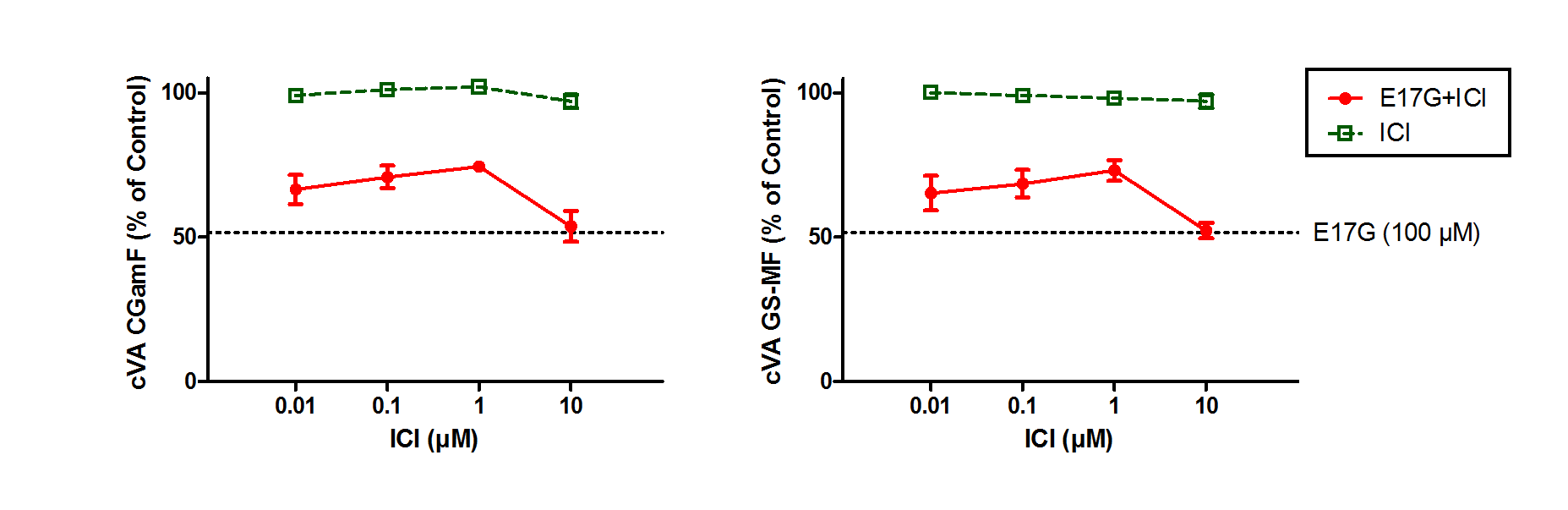

Supplement: Figure S1 — Determination of maximal prevention by ICI 182,780 (ICI) against E17G-induced impairment of canalicular vacuolar accumulation (cVA) of CGamF (left panel) and GS-MF (right panel). IRHC were preincubated with ICI (0.01–10 µM) for 15 minutes, and then exposed to E17G (100 µM) or DMSO (Control) for an additional 20-min period. cVAs of CGamF and GS-MF were calculated as the percentage of couplets displaying visible fluorescence in their canalicular vacuoles from a total of at least 200 couplets per preparation, and expressed as percentage of control cVA values. Dotted line represents the cVA of IRHC exposed to E17G (100 µM) alone. ICI itself did not induce any changes in cVA of CGamF and GS-MF. Data are expressed as mean ± SEM (n = 3). (ZIP) [file pone.0050711.s001.zip › Fig S1.tif]
